# The “blownknee” patient’s stress fracture of distal tibial component after unilateral TKA: A case report

**DOI:** 10.1097/MD.0000000000039382

**Published:** 2024-08-23

**Authors:** Quanxiang Sun, Changjie Liu, Xuedong Sun, Zezhong Liu, Xiaoguang Liu, Wei Li, Yimin Zhang

**Affiliations:** a School of Clinical Medicine, Shandong Second Medical University, Weifang, Shandong, China; b Department of Orthopedics, Weifang Second People’s Hospital, Weifang, Shandong,China; c Department of Joint Surgery, The First Affiliated Hospital of Shandong Second Medical University, Weifang, Shandong, China.

**Keywords:** blownknee, lower limb force line, ORIF, PPF, TKA, walking gait, weight-bearing.

## Abstract

**Rationale:**

Periprosthetic fractures (PPF) are rare complications of total knee arthroplasty (TKA). The most common PPF after TKA is supracondylar femoral fracture, which is a relatively rare complication that is usually associated with high-energy trauma, with a reported incidence ranging from 0.4 to 1.7% according to the AOANJRR. However, in TKA patients, it is rarer that the stress fracture around the tibial prosthesis occurs due to changes in the lower limb force line, increasing weight-bearing, and changes in walking gait.

**Patient concerns:**

A 68-year-old woman visited our hospital with “both knees had aggravated pain and deformity for 8 years.” TKA was performed first on the left knee and the patient was discharged within 1 week. Three months later, the patient complained of pain in the upper middle 1/3 part of the medial tibia for 2 weeks, which gradually worsened and affected weight-bearing.

**Diagnoses:**

Physical examination showed that the left knee joint presented varus deformity, and the right valgus deformity, which diagnosed as osteoarthritis of both knees and was so-called “blownknee”. The disease was initially diagnosed as osteoarthritis of both knees on first admission and PPF of the tibia in second.

**Interventions:**

Three operations were performed on this patient. The first was TKA of the left knee, the second was open reduction and internal fixation of the PPF of the tibia 3 months after the first operation, and the third was TKA of the right knee.

**Outcomes:**

Until now, the patient has had no recurrent PPF, and the fracture is healing from the last X-ray.

**Lessons:**

Clinicians should be aware of the possibility of PPF after TKA, especially in such patients, the most preferred surgical treatment method was open reduction and internal fixation of fractures using locking plates, and if the PPF with loosened implants, Revision TKA, or megaprosthesis was the better choice.

## 1. Introduction

The concept of “blownknee” is a phenomenon of valgus deformity of unilateral knee joint against varus one of its counterpart in same person. We reported a rare case of “blownknee” with ipsilateral distal tibial periprosthetic fracture (PPF) after total knee arthroplasty (TKA), the reason of which might be the change of force line from varus to normal, engendering stress increasing in medial tibial cortex at the distal end of prosthesis. Meanwhile, the weight burden increase by the left lower limb led to a more concentrated stress at the distal end of the tibial extension rod.

## 2. Case presentation

**Figure 1.**
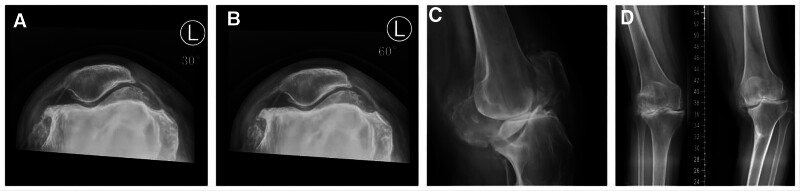
A sign of stress fracture was found in the left medial proximal tibia by X-ray examination. ((A and B) Left knee flexion = 30° and 60°. (C) Side position of the left knee joint. (D) **→** represents the stress fracture in the left knee joint).

**Figure 2.**
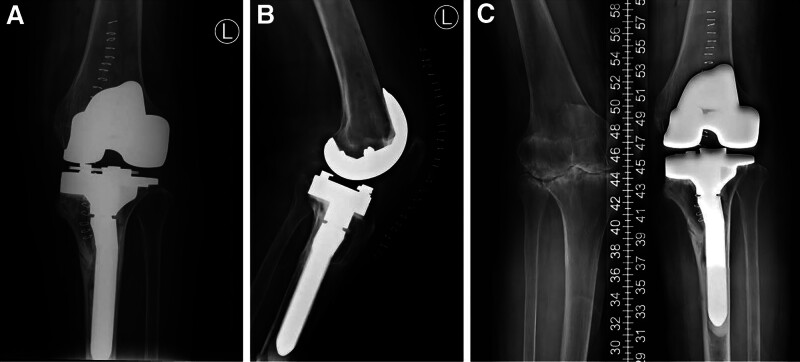
TKA was performed first on the left knee using a cemented rotating-hinge prosthesis successfully.

A 68-year-old female patient (height, 160 cm; weight, 56 kg; body mass index, 21.875) was admitted on February 14th, 2023 due to severe osteoarthritis of both knees. Blood tests revealed a normal osteoporosis index. Physical examination revealed valgus deformity in the right knee joint and varus deformity in the left knee. A sign of stress fracture was found in the left medial proximal tibia by X-ray examination (Fig. 1). TKA was performed first on the left knee using a cemented rotating-hinge prosthesis successfully, and weight-bearing and walking were performed 2 days later. The patient was discharged from the hospital 1 week postoperative (Fig. 2).

**Figure 3.**
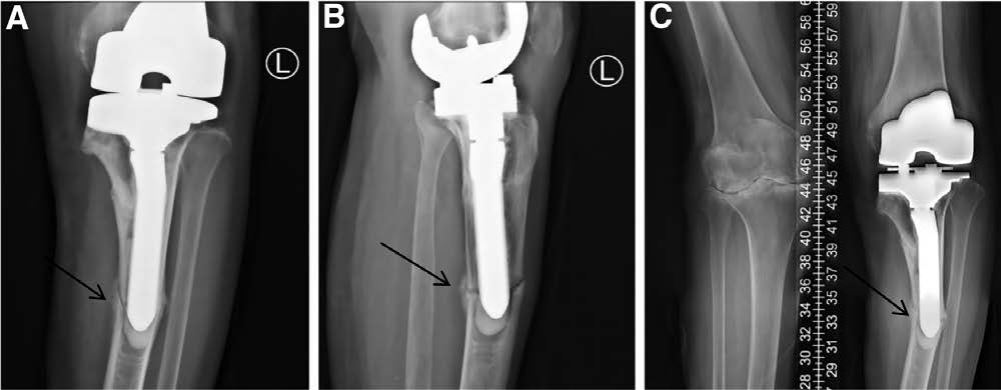
X-ray diagnosis of the tibia fracture of the middle and upper parts, slightly more distal to the original preoperative stress fracture, without any trauma. (**→** represents the PPF in the left knee joint).

The patient was readmitted over 3 months after the first TKA, complaining of pain in the anteromedial part of the middle and upper segments of the left tibia for 2 weeks, which gradually worsened and affected weight-bearing. Clinical manifestation combined with X-ray diagnosis of the tibia fracture of the middle and upper parts, slightly more distal to the original preoperative stress fracture, without any trauma (Fig. 3).

**Figure 4.**
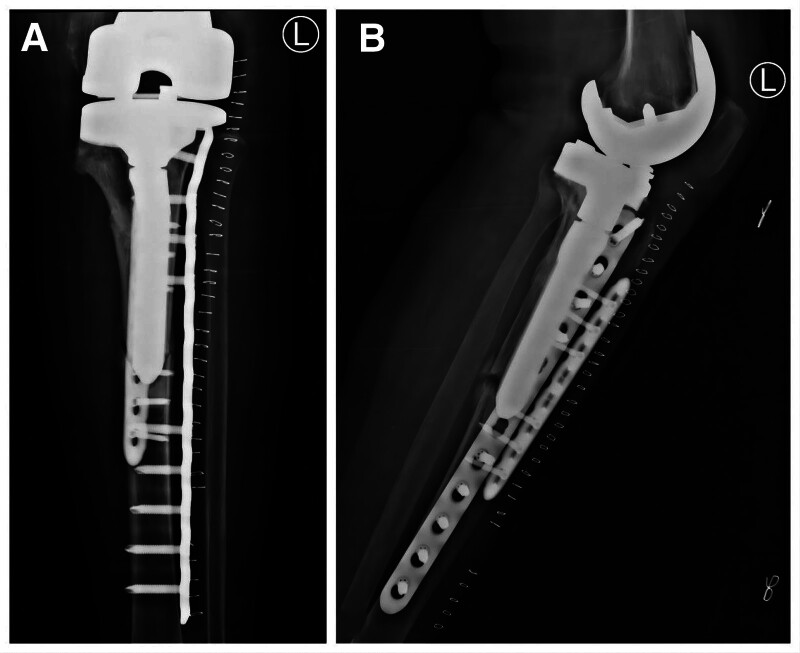
Open reduction and internal fixation applying 2 plates was implemented for the fracture.

Open reduction and internal fixation (ORIF) applying 2 plates was implemented for the fracture, and that pain disappeared postoperatively. Furthermore, for both the rehabilitation well (Fig. 4).

**Figure 5.**
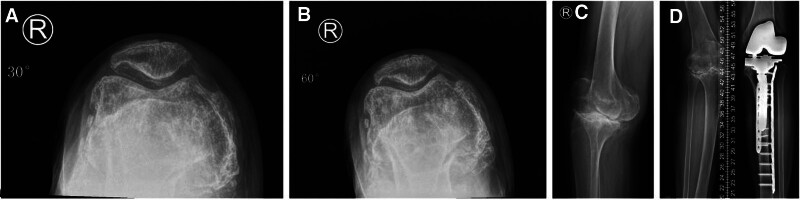
The X-ray was taken 3 months after the surgery. ((A and B) Left knee flexion = 30° and 60°. (C) Side position of the right knee joint. (D) The X-ray of bilateral knee joint.).

**Figure 6.**
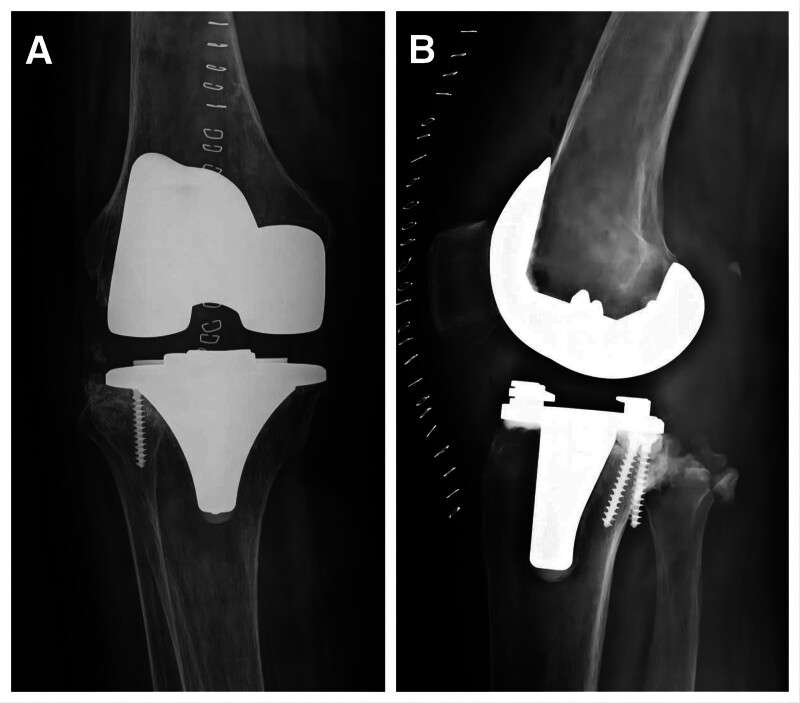
Aiming to balance of bilateral lower limbs, TKA was implemented on the right knee 3 months after the surgery.

Aiming to balance of bilateral lower limbs, TKA was implemented on the right knee 3 months later (Figs. 5 and 6).

**Figure 7.**
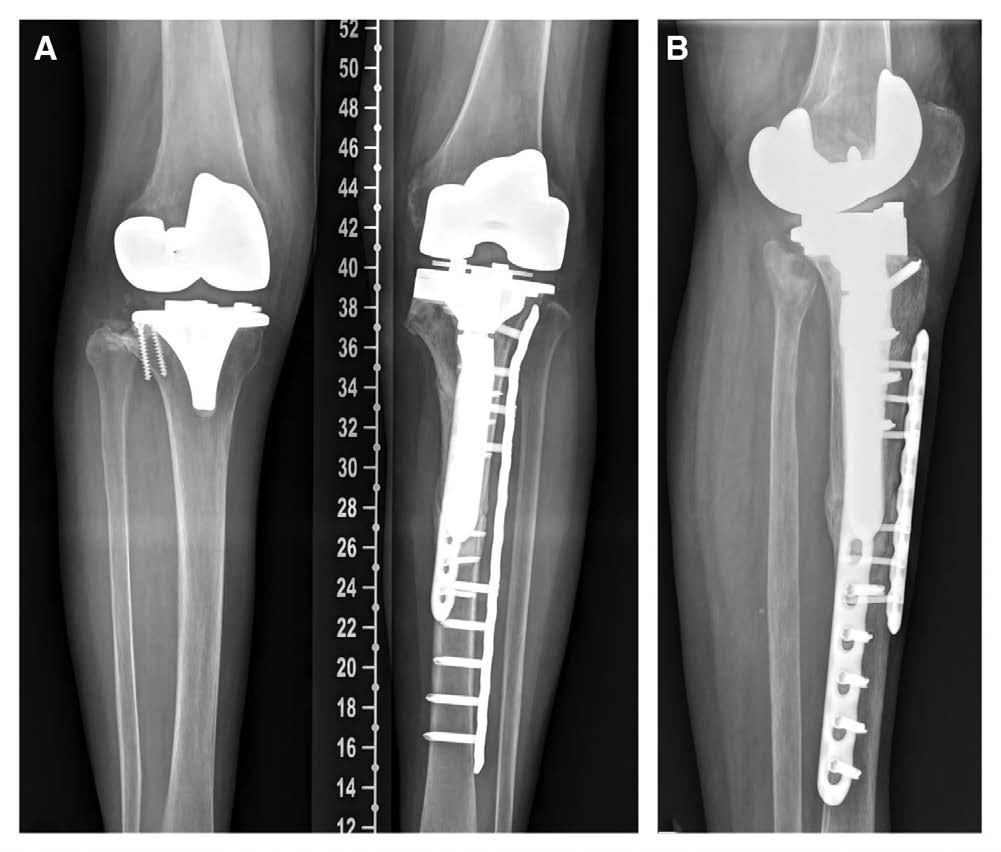
Until now, the patient has had no recurrent PPF, and the fracture is healing from the last X-ray.

Until now, the patient has had no recurrent PPF, and the fracture is healing from the last X-ray (Fig. 7).

## 3. Discussion

Complications after TKA include deep vein thrombosis, prosthetic infection, massive bleeding, wound liquefaction, and peripheral nerve injury.^[1,2]^ PPF are more rare complication, often associated with high-energy trauma, with a reported incidence ranging from 0.4% to 1.7% according to the AOANJRR.^[3–6]^ The concept of PPF is fracture around the implant (metal or other). PPF associated with TKA is defined as fractures around the knee joint (femur, tibia, or patella) occurring within 5 cm of the intramedullary stem of the prosthesis or 15 cm of the knee joint.^[7]^ Rocca GJ et al reported that PPF is a rare complication of TKA and is relatively common in revision surgery.^[8]^ Most researchers have pointed out that PPF is mainly caused by direct or indirect violence, which can be clearly described by patients with accidental trauma.^[9,10]^ In addition, periprosthetic infection, prosthesis loosening, stress force line change, reduced periprosthetic bone density, osteoporosis, prolonged steroid use, rheumatoid arthritis, and excessive BMI are also high-risk factors for knee PPF.^[4,7,11,12]^ In this study, almost all other risk factors such as trauma, BMI, osteoporosis, and infection were excluded. In this case, no prosthetic position changed and loosening occurred, and measurement of the cortical thickness in the fractured part between pre- and postoperation showed no significant discrepancy.^[4,8,10,11,13]^

“Blownknee,” as the name implies, refers to that 1 knee joint was valgus deformity, meanwhile the other was varus deformity. The patient in this study had a long history of “blownknee,” severe valgus deformity in the right knee, and varus in the left knee, causing the stress point to concentrate inward prior to the primary TKA of the left knee. Following TKA, the force line was corrected and a reverse pulling tension against the medial stress point yielded, especially when walking with the contralateral valgus knee, giving rise to another stress fracture.

Generally, based on the above analysis, there might be factors for this PPF: ① correction of the left lower limb after TKA, resulting in the stress point in the middle and upper segments of the left tibia, which produced outward tension. ② Because the force line returned to normal, the weight burden on the left knee was larger than before walking.

PPF can be treated with ORIF or revision total knee arthroplasty. This patient’s Felix classification of the tibial fracture is type IIIA, and for type IIIA fractures, a long locking plate is usually required to stabilize the fracture and provide stable fixation.^[9,10,14]^ Michael P. Morwood et al recommend double plate for proximal tibial fractures and single plate or nail for intermediate and distal fractures.^[9]^ Considering the first operation by the cemented rotating-hinge prosthesis, no prosthesis loose, and right knee valgus deformity, we selected double-plate fixation to correct the fracture and enhance the bearing force of the left tibia.

In this case, we should reflect on the measures that should be taken to avoid this complication. If the patient has a “blownknee,” whether we can let the patient accept the opposite TKA in a short term to correct the excessive loading of the patient, whether we can delay the time of underground walking after surgery, If the patients simultaneously have the knee joint peripheral infection, knee prosthesis loosening, periprosthetic bone density reduced, osteoporosis and excessive BMI, how to reduce the risk of the PPF is the significant problem. Yang et al discovered that robotic-assisted total knee arthroplasty can better correct severe varus/valgus deformity of the knee joint,^[7]^ whether this can reduce the risk of PPF requires further confirmation.

In conclusion, we should keep this rare complication in mind and prevent further complications. For PPF, the most preferred surgical treatment method was ORIF of fractures using locking plates. And if the PPF with loosened implants, revision total knee arthroplasty, or megaprosthesis was the better choice, Koji Nozaka et al reported the first case in which Ilizarov external fixation was used for a periprosthetic tibial fracture after TKA and achieved positive results.^[7,15–18]^ However, because the follow-up period of the right knee is only 4 months, there is insufficient evidence to prove that the patient will not undergo PPF of the right knee joint in the future; therefore, to prevent another PPF, we will continuously pay attention to the patient’s postoperative recovery condition.

## 4. Conclusions

Here, we present a rare case of PPF after TKA. Clinicians should be aware of the complications associated with TKA. Especially, like this “blownknee” patient, after we perform the TKA, we should evaluate the lower limbs condition after surgery, whether the patient can habituated to the change in the lower limb force line, whether the patient can able to walk with full-weight bearing, whether an anti-osteoporosis therapy is required, and so on. Once patients develop postoperative complications, we should actively choose an appropriate treatment plan.

## Acknowledgments

The authors gratefully acknowledge the patients who agreed to participate in the study.

## Author contributions

**Conceptualization:** Xuedong Sun, Wei Li.

**Formal analysis:** Xuedong Sun.

**Investigation:** Xuedong Sun.

**Resources:** Changjie Liu.

**Software:** Zezhong Liu.

**Validation:** Xiaoguang Liu.

**Visualization:** Xiaoguang Liu.

**Writing – original draft:** Quanxiang Sun.

**Writing – review & editing:** Wei Li, Yimin Zhang.
